# *Streptococcus pneumoniae* genomic datasets from an Indian population describing pre-vaccine evolutionary epidemiology using a whole genome sequencing approach

**DOI:** 10.1099/mgen.0.000645

**Published:** 2021-09-08

**Authors:** Geetha Nagaraj, Vandana Govindan, Feroze Ganaie, V. T. Venkatesha, Paulina A. Hawkins, Rebecca A. Gladstone, Lesley McGee, Robert F. Breiman, Stephen D. Bentley, Keith P. Klugman, Stephanie W. Lo, K. L. Ravikumar

**Affiliations:** ^1^​ Central Research Laboratory, Kempegowda Institute of Medical Sciences, Bangalore, India; ^2^​ Hubert Department of Global Health, Rollins School of Public Health, Emory University, Atlanta, GA, USA; ^3^​ Parasites and Microbes, Wellcome Sanger Institute, Hinxton, UK; ^4^​ Centers for Disease Control and Prevention, Atlanta, GA, USA

**Keywords:** global pneumococcal sequence cluster, genomic dataset, India, pre-vaccine, *S. pneumoniae*

## Abstract

Globally, India has a high burden of pneumococcal disease, and pneumococcal conjugate vaccine (PCV) has been rolled out in different phases across the country since May 2017 in the national infant immunization programme (NIP). To provide a baseline for assessing the impact of the vaccine on circulating pneumococci in India, genetic characterization of pneumococcal isolates detected prior to introduction of PCV would be helpful. Here we present a population genomic study of 480 *Streptococcus pneumoniae* isolates collected across India and from all age groups before vaccine introduction (2009–2017), including 294 isolates from pneumococcal disease and 186 collected through nasopharyngeal surveys. Population genetic structure, serotype and antimicrobial susceptibility profile were characterized and predicted from whole-genome sequencing data. Our findings revealed high levels of genetic diversity represented by 110 Global Pneumococcal Sequence Clusters (GPSCs) and 54 serotypes. Serotype 19F and GPSC1 (CC320) was the most common serotype and pneumococcal lineage, respectively. Coverage of PCV13 (Pfizer) and 10-valent Pneumosil (Serum Institute of India) serotypes in age groups of ≤2 and 3–5 years were 63–75 % and 60–69 %, respectively. Coverage of PPV23 (Merck) serotypes in age groups of ≥50 years was 62 % (98/158). Among the top five lineages causing disease, GPSC10 (CC230), which ranked second, is the only lineage that expressed both PCV13 (serotypes 3, 6A, 14, 19A and 19F) and non-PCV13 (7B, 13, 10A, 11A, 13, 15B/C, 22F, 24F) serotypes. It exhibited multidrug resistance and was the largest contributor (17 %, 18/103) of NVTs in the disease-causing population. Overall, 42 % (202/480) of isolates were penicillin-resistant (minimum inhibitory concentration ≥0.12 µg ml^−1^) and 45 % (217/480) were multidrug-resistant. Nine GPSCs (GPSC1, 6, 9, 10, 13, 16, 43, 91, 376) were penicillin-resistant and among them six were multidrug-resistant. Pneumococci expressing PCV13 serotypes had a higher prevalence of antibiotic resistance. Sequencing of pneumococcal genomes has significantly improved our understanding of the biology of these bacteria. This study, describing the pneumococcal disease and carriage epidemiology pre-PCV introduction, demonstrates that 60–75 % of pneumococcal serotypes in children ≤5 years are covered by PCV13 and Pneumosil. Vaccination against pneumococci is very likely to reduce antibiotic resistance. A multidrug-resistant pneumococcal lineage, GPSC10 (CC230), is a high-risk clone that could mediate serotype replacement.

## Data Summary

Genome sequences are deposited at the European Nucleotide Archive (ENA) and the accession number is available in the metadata and study accession is PRJEB3084. The phylogenetic snapshot is available at https://microreact.org/project/GPS_India. The authors confirm all supporting data, code and protocols have been provided within the article or through supplementary data files.

**Impact Statement**

This study provides a detailed report of the population genetic structure of a collection of pneumococcal disease and carriage isolates from children and adults in India. It provides genomic data to understand the prevalence of serotypes, pneumococcal lineages and antimicrobial resistance prior to vaccine introduction, so as to enable future studies to assess these changes after the roll out of vaccines. This study also highlights a high-risk clone, GPSC10 (CC230), that could potentially evade PCV13. The current findings demonstrate the usefulness of genomic surveillance in understanding the pneumococcal epidemiology and evolution so as to inform disease prevention.

## Background

*Streptococcus pneumoniae* is a human nasopharyngeal commensal and a respiratory pathogen causing a spectrum of diseases ranging from mild respiratory illness (e.g. otitis media) to severe diseases (e.g. pneumonia and meningitis) [1]. In 2015, India was estimated to have the highest burden of pneumococcal deaths [2]; India, Nigeria, Democratic Republic of the Congo and Pakistan accounted for 50 % of all pneumococcal deaths. In India, 68 700 [uncertainty range (UR) 44600–86 000] pneumococcal deaths were estimated to have occurred in children aged 1–59 months. Severe pneumococcal disease in India manifests primarily as severe pneumonia. There were 1·6 million (UR 1·2–1·8) estimated cases of severe pneumococcal pneumonia in 2015, accounting for more than 97 % of all severe pneumococcal disease [2]. The recent roll-out (May 2017) of pneumococcal conjugate vaccine (PCV) in the national infant immunization schedule is expected to contribute to reductions in this disease burden [3]. Pneumococcal vaccination is not recommended for healthy adults under the age of 65 years in India. Vaccination with PPV23 in adults above 65 years of age is recommended because of the overall higher incidence of invasive pneumococcal disease in this age group [3–7].

Changes in epidemiology and population structure are likely to follow vaccine introduction [4]. Understanding the changes requires reproducible and robust molecular typing methods. Molecular typing of *S. pneumoniae* helps to delineate the genetic structure of bacterial populations and infer evolutionary relationships between isolates. Whole-genome sequencing (WGS) with its high discriminatory power has become a feasible tool for bacterial typing, given steadily decreasing associated costs [8, 9].

With 20 027 pneumococcal genomes sequenced, the Global Pneumococcal Sequencing project (GPS, http://www.pneumogen.net/gps/) defined 621 pneumococcal lineages, named Global Pneumococcal Sequence Clusters (GPSCs). This has contributed to the increased understanding of the pneumococcal population structure globally and provided further information on the distribution of serotypes and antibiotic resistance [10]. As part of the GPS project, we analysed the WGS data of invasive and carriage pneumococcal isolates (*n*=480) from Indian adults and children over an 8 year period (2009–2017) before the introduction of 13-valent PCV (PCV13) in the national infant immunization programme (NIP). Serotype distribution, antibiotic resistance and capsular switching in a sample of pneumococcal isolates is reported and discussed.

## Methods

### Pneumococcal isolates

We collected pneumococcal isolates from 14 regions in India through PNEUMONET [11] and the multicentric PIDOPS project [12] between 2009 and 2017. PNEUMONET and the PIDOPS project targeted routine collection of disease and carriage pneumococcal isolates among all age groups across sentinel sites. The isolates were collected prior to the introduction of PCV13 (May 2017) in the NIP. The isolates were stored in Central Research Laboratory, KIMS, Bangalore, for further analyses.

**Fig. 1.**
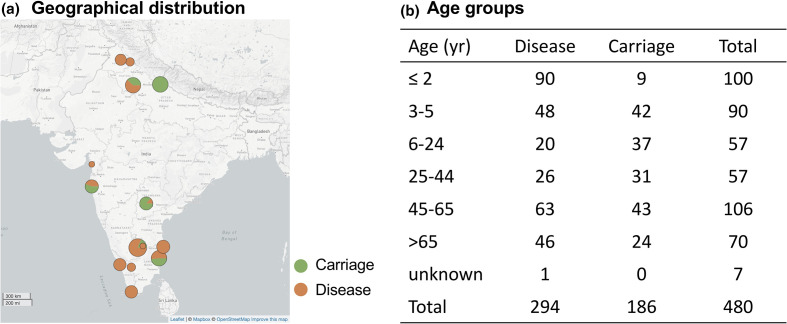
The geographical and age distribution of pneumococcal collection in this study. (**a**) The size of the circle is proportionate to the number of isolates; breakdown is by carriage and disease. (**b**) Age-wise distribution of disease and carriage pneumococcal isolates.

The collection consisted of 480 pneumococcal isolates, including carriage isolates (*n*=186) and disease isolates (*n*=294) (Fig. 1, Table S1, Table S8, available in the online version of this article). The disease isolates were collected across all sampling sites except for Kharagpur while carriage isolates were from Bangalore, Delhi, Hyderabad, Kharagpur, Mumbai and Pondicherry. Of isolates causing disease, they were recovered from blood culture (*n*=226), cerebrospinal fluid (*n*=36), pleural fluid (*n*=7) and other sources such as eye swabs, ascitic fluid and lung abscess (*n*=25).

### Genome sequencing and analyses

The pneumococcal isolates were subject to WGS on an Illumina Hi-Seq platform to produce paired-end reads with an average of 151 bases in length and the raw data were deposited in the ENA (Table S8). WGS data were processed as previously described [10]. Briefly, we derived the serotype using SeroBA [13] and multilocus sequence types (MLSTs) using MLSTcheck [14]. We defined MLST clonal complexes (CC) as STs with single locus variant (SLV) differences, within the GPS dataset as previously described [10]. Antibiotic resistance profiles and presence of pili were predicted using the CDC pipeline from genome data [15–18]. The CDC pipeline script and reference database are deposited at https://github.com/BenJamesMetcalf/Spn_Scripts_Reference. Both *in silico* prediction of serotypes and antibiotic resistance were compared with available phenotypic testing results and showed high concordance [10]. Multidrug resistance (MDR) was defined as isolates resistant to three or more classes of antibiotics. The population structure was defined by assigning GPSC to each isolate using a Kmer-based clustering method, PopPUNK [19], and a reference list of pneumococcal genomes (*n*=34780) that is available at https://www.pneumogen.net/gps/assigningGPSCs.html. Phylogenetic analysis was performed on all isolates by constructing a maximum-likelihood tree using FastTree version 2.1.10, which used heuristics to restrict the search for better trees and estimates a rate of evolution for each site [20]. The phylogeny was based on SNPs extracted from an alignment generated by mapping reads to the reference genome of *S. pneumoniae* ATCC 700669 (NCBI accession number FM211187) using Smalt, version 0.7.4, with default settings [21]. The metadata and analysis results can be interactively visualized online using Microreact at https://microreact.org/project/GPS_India.

### Capsular switching

Histories of capsular switching were inferred in the isolates with identical ST but different serotypes in the Indian dataset in this study. For each ST, we then examined the genetic relatedness of isolates in a lineage-specific phylogeny. The lineage-specific phylogeny was constructed using GPS published isolates belonging to this ST and other related STs within a GPSC [22]. Including the GPS isolates from other countries provided a global context so as to better understand if the observation was a result of (1) an in-country capsular switching (isolates from India clustered together in the global phylogeny) or (2) importations of isolates with identical ST but different serotype from other countries (isolates from India did not cluster together but clustered with isolates from other countries). In brief, for each GPSC, the lineage-specific phylogeny was built from a recombination-free SNP alignment. This alignment was created by first mapping reads to a lineage-specific reference genome using Burrows Wheeler Aligner version 0.7.17-r1188 (BWA), then removing recombination regions, and extracting SNPs for tree reconstruction using GUBBINS version 2.4.1 [23,24].

### Definitions and statistical analyses

Serotypes were grouped into two categories: (1) vaccine serotype (VT) which included PCV13 serotypes 1, 3, 4, 5, 6A, 6B, 7F, 9V, 14, 18C, 19A, 19F and 23F; and (2) non-vaccine serotype (NVT) which included serotypes not in PCV13. Differences in prevalence of antibiotic resistance and serotypes were detected by Fisher’s exact test. Two-sided *P* values of <0.05 were considered statistically significant. Multiple testing was adjusted using the Benjamini–Hochberg false discovery rate of 5 %. The statistical analysis was carried out in R version 3.5.2 and R scripts used for analyses were deposited at https://github.com/StephanieWLo/Genomic-Surveillance

## Results

### Prevalence of pneumococcal serotypes

Serotypes as predicted from WGS data revealed 54 serotypes plus one isolate identified as non-typeable. Stratified by carriage and disease, the number of serotypes were 41 and 48, respectively. Thirteen serotypes (1, 7F, 8, 20, 33F, 2, 38, 12F, 27, 45, 25A, 25F, 9L, decreasing order of prevalence) were only found among diseased isolates while seven serotypes (6D, 7C, 19B, 48, 9N, 18B, nontypeable, descending prevalence) were only detected among carriage isolates. Examination of the *cps* region (flanked by *dexB* and *aliA*) of the non-typeable isolate showed that there is an insertion of a surface protein NspA, which was previously described by Salter *et al*. [25]. There was no significant difference in serotype prevalence between children aged under 5 years and adults aged 50 and above (Tables S6 and S7).

**Fig. 2.**
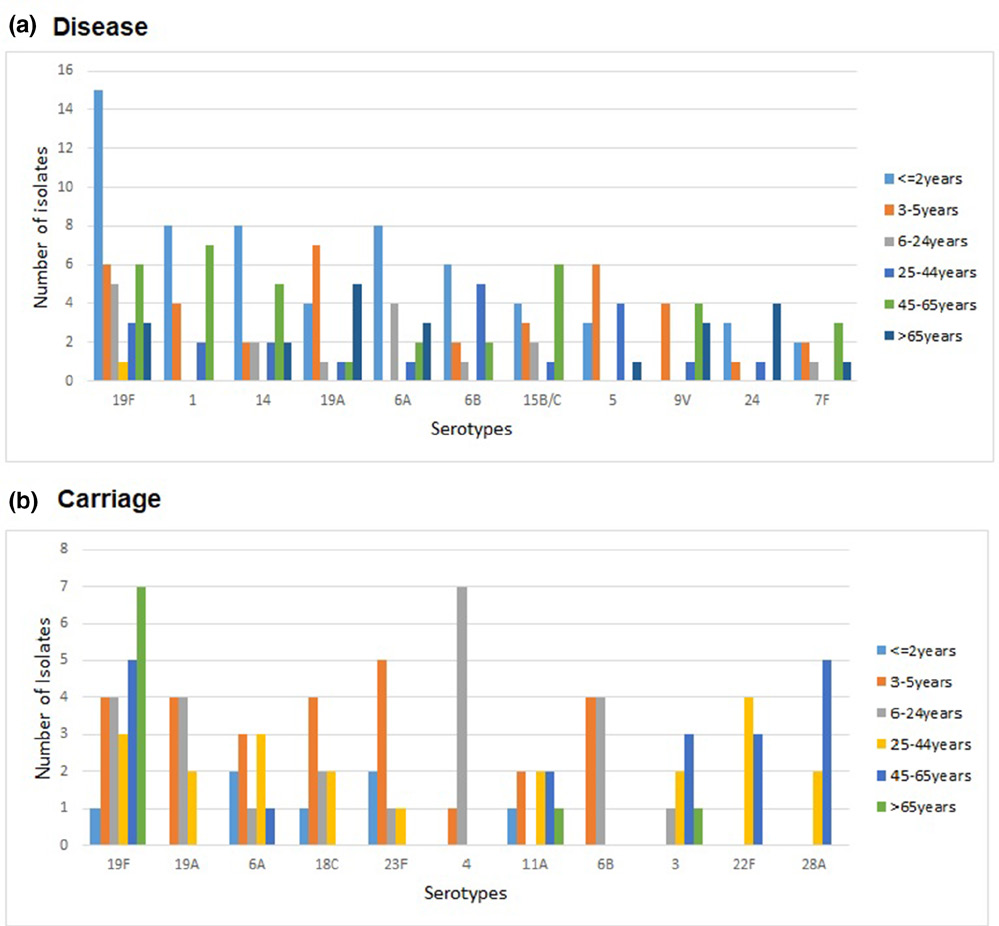
The top ten serotypes among pneumococci from (a) disease-causing and (b) carriage populations, stratified by age groups.

The top ten serotypes were PCV13 serotypes, except for serotype 15B/C and 24A/F among disease isolates and except for 11A, 22F and 28A in carriage isolates (Fig. 2). Overall, serotype 19F was the most prevalent serotype among both disease (13 %, 38/294) and carriage (13 %, 24/186) isolates. It is of note that serotype 4, which was significantly associated with high invasive disease potential [10], was found to be carried by eight children aged <9 years in the Pondicherry area in 2014. They all belonged to a single ST205 (GPSC27), suggesting local clonal transmission. Four serotype 4 were also detected in disease isolates in Pondicherry (*n*=1, GPSC27) and Bangalore (*n*=3, GPSC86). Rare, but invasive, serotypes such as serotype 2 (GPSC96/CC74, *n*=2) and serotype 45 (GPSC245/ST3022, *n*=1) were observed to cause disease in India among this sampling of isolates. Other NVTs with high invasive disease potential [10] such as serotype 8 (GPSC336/CC12793 and GPSC227/CC10588, *n*=8), 12F (GPSC26/CC989, *n*=2) and 33F (GPSC236/CC14568, *n*=3) were also detected among disease isolates.

Vaccine coverage by age group and clinical manifestation are summarized in Table 1. The predicted vaccine coverage against disease-causing isolates in children aged ≤2 years of age in this study was 57/90 (63 %) for PCV13, 54/90 (60 %) for the ten-valent Pneumosil manufactured by Serum Institute of India, 52/90 (58 %) for the GSK PCV10 vaccine (1, 4, 5, 6B, 7F, 9V, 14, 18C, 19F and 23F), 58/90 (64 %) for the Merck PCV15, 62/90 (69 %) for the Pfizer PCV20 vaccine and 67/90 (74 %) for the Merck PCV24 vaccine. The latter three vaccines are still under development. Of the 90 disease isolates from children aged 2 years or under, the prevalent NVTs were 15B/C (*n*=4), 16F (*n*=4) and 24A/F (*n*=3), and one or two isolates for other 19 NVTs. The major NVTs in different age groups are summarized in Table S4 and Fig S1.

**Table 1.** The top five serotypes and vaccine coverage by age groups

	Disease (%)				Carriage (%)			
Age group (years)	*n*	Pneumosil*	PCV13*	Top 5 serotypes	*n*	Pneumosil	PCV13	Top 5 serotypes
≤2	90	54 (60)	57 (63)	19F, 1, 14, 6A, 6B	9	5 (56)	6 (67)	6A, 23F
3–5	48	33 (69)	36 (75)	19F, 19A, 5, 1, 9V	42	21 (50)	26 (62)	18C, 19A, 23F, 6B, 6A
6–24	20	14 (70)	16 (80)	19F, 6A, 14, 6B, 15B/C	37	15 (40)	25 (67)	4, 19F, 19A, 6B, 6D
25–44	26	20 (77)	20 (77)	6B, 5, 19F, 1, 14	31	10 (32)	14 (45)	22F, 6A, 19A, 19F, 18C
45–65	63	31 (49)	36 (57)	1, 15B/C, 19F, 8, 14	43	9 (21)	12 (28)	19F, 28A, 17F, 3, 22F
>65	46	20 (43)	25 (54)	19A, 19F, 24, 3, 6A, 9V	24	10 (42)	11 (46)	19F, 35A, 13, 14

*Pneumosil (Serum Institute of India, vaccine includes serotypes 1, 5, 6A, 6B, 7F, 9V, 14, 19A, 19F and 23F) and PCV13 (Pfizer, vaccine includes serotypes 1, 3, 4, 5, 6A, 6B, 7F, 9V, 14, 18C, 19A, 19F and 23F) contain serotype 6B. Serotypes with one or no isolates are not listed. The non-PCV13 serotypes are underlined.

Among disease-causing isolates from the ≥50 years age group in this study, the predicted vaccine coverage was 67/94 (71.2 %) for PPV23 and 54/94 (57.4 %) for PCV13 vaccine. 24A/F, 15A and 6A were the major non-PPV23 serotypes in this age group (Table S5, Fig. S2).

### Prevalence of pneumococcal lineages

Our results showed that pneumococcal isolates from India have a diverse genetic background represented by 110 GPSCs and 223 STs (103 CCs and 50 singletons). Among them, there are 20 novel GPSCs (*n*=42) and 75 STs (*n*=114) (see metadata for more details). Of the 20 novel GPSCs, they are mainly observed in India with only five also found in neighbouring countries Bangladesh (GPSC559, 651 and 655) and Nepal (GPSC652 and 712) (GPS database last accessed in November 2020). These regional lineages mainly expressed non-PCV13 serotypes (62 %, 26/42) and were usually found in the carriage population (62 %, 26/42). In contrast, six of eight globally spreading lineages recognized in the previous GPS study [10] were found in the current collection with a prevalence of 10.8 % (GPSC1/CC320, *n*=52), 2.5 % (GPSC6/CC156/Spain9V-3, *n*=12), 2.1 % (GPSC16/CC81/Spain23F, *n*=10), 1.3 % (GPSC32/CC218, *n*=6), 0.8 % (GPSC23/CC385, *n*=4) and 0.2 % (GPSC12/CC505/Netherlands3-31, *n*=1). GPSC7 (CC439) and GPSC18 (CC15/England14-9) were not detected.

**Fig. 3.**
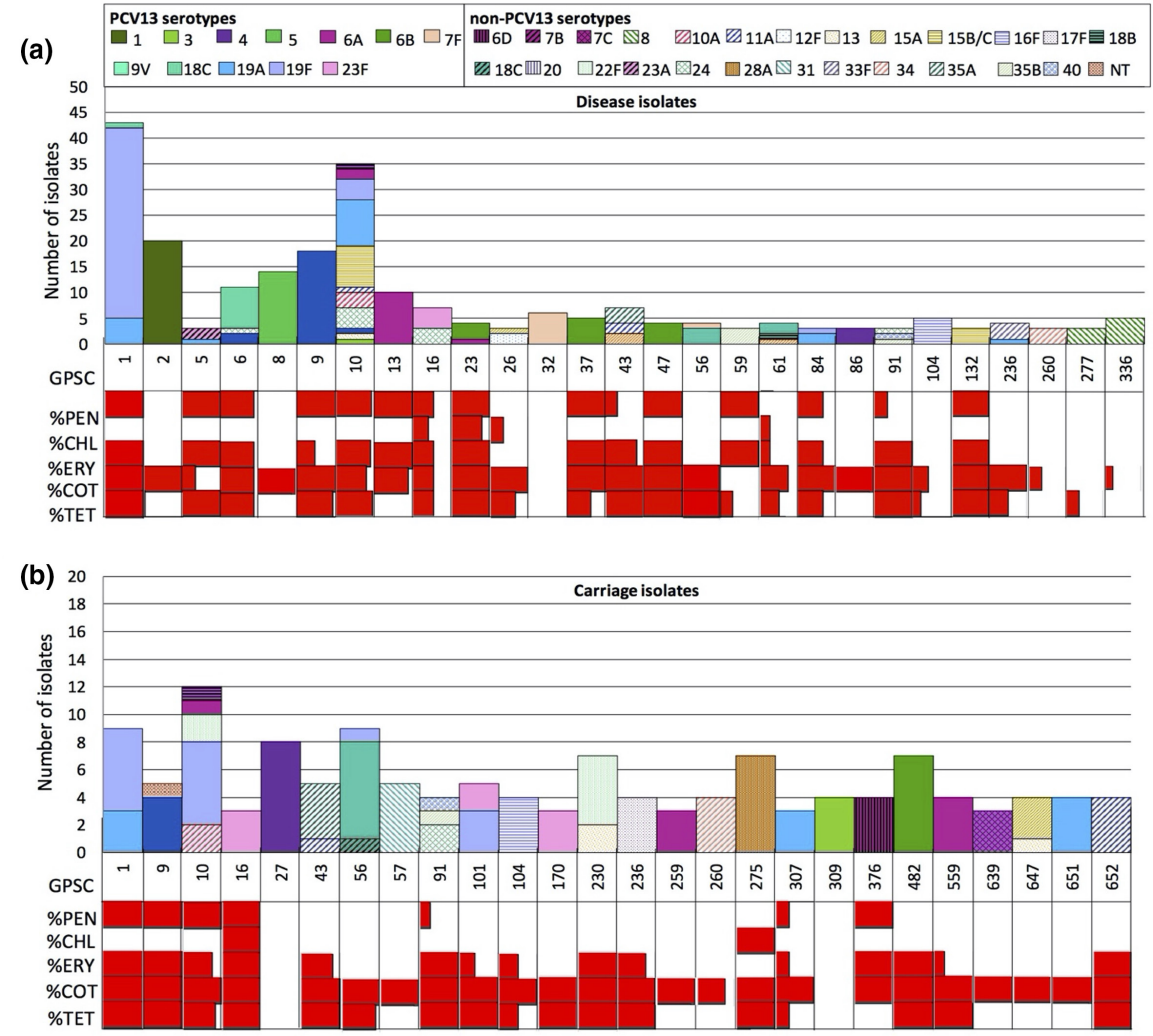
(a) Serotype composition and prevalence of antibiotic resistance by Global Pneumococcal Sequence Clusters (GPSCs) among disease (*n*=294) and (b) carriage (*n*=186) isolates from India. Lineages with fewer than three isolates are not shown. PCV13 serotypes are indicated in solid colour and non-PCV13 serotypes in colours with patterns. This figure shows that GPSC10 is one of the major pneumococcal lineages in both populations. It expresses a variety of serotypes and is multidrug resistant. Red=resistant; white=sensitive.

The top five pneumococcal lineages and their associated serotypes are summarized in Table 2. GPSC1 (CC320) was the top lineage in both carriage and disease-causing populations and accounted for 50 % (12/24) and 82 %(31/38) of the most prevalent serotype 19F, respectively. The top five lineages causing disease mainly expressed PCV13 serotypes, except for GPSC10 (CC230/Denmark14-32) (serotype 15B/C, 24, 10A, 13, 7B, 11A). GPSC10 was the largest contributor (17 %, 18/103) of NVTs in the disease-causing population and accounted for 50 % (8/16) of the major non-PCV13 serotype 15B/C. Of the 106 GPSCs found in this study, 59 GPSCs (55 %) were NVT lineages, 38 (36 %) were VT lineages and nine (8.5 %) were lineages (GPSC 5, 6, 9, 10, 16, 56, 61, 236, 376) with both VT and NVT isolates (Fig. 3).

**Table 2.** The five most prevalent pneumococcal lineages and their associated serotypes in a collection of disease (*n*=294) and carriage (*n*=184) isolates from India

	Disease (*n*=294)			Carriage (*n*=186)		
Rank	GPSC (major CC)	*N* (%)	Associated serotypes	GPSC (major CC)	*N* (%)	Associated serotypes
1st	GPSC1 (CC320)	37 (13)	19F (*n*=31), 19A (*n*=5), 9V (*n*=1)	GPSC1 (CC320)	15 (8)	19F (*n*=12), 19A (*n*=3)
2nd	GPSC10 (CC230)	35 (12)	19A (*n*=9), 15B/C (*n*=8), 24F (*n*=4),19F (*n*=4), 10A (*n*=3), 6A (*n*=2),3 (*n*=1), 13 (*n*=1), 14 (*n*=1), 7B (*n*=1), 11A (*n*=1)	GPSC10 (CC230)	12 (6)	19F (*n*=6), 10A (*n*=2), 22F (*n*=2), 6A (*n*=1), 7B (*n*=1)
3rd	GPSC2 (CC217)	20 (7)	1 (*n*=20)	GPSC56 (CC5068)	10 (5)	18C (*n*=8), 18B (*n*=1), 19F (*n*=1)
4th	GPSC9 (CC63)	18 (6)	14 (*n*=18)	GPSC27 (CC205)	8 (4)	4 (*n*=8)
5th	GPSC8 (CC289)	14 (5)	5 (*n*=14)	GPSC230 (CC2754)	7 (4)	22F (*n*=5), 13 (*n*=2)
6th	–	–	–	GPSC275 (CC1853)	7 (4)	28A (*n*=7)
7th	–	–	–	GPSC482 (CC13588)	7 (4)	6B (*n*=7)

The non-PCV13 serotypes are underlined. GPSC, global pneumococcal sequencing cluster; CC, clonal complex.

### Capsular switching and pilus

Six STs were found to express different serotypes in this dataset and a GPSC-specific phylogeny of each of these corresponding STs was built to place the Indian isolates in global context (Table 3). Overall, pilus islet 1 (PI-1) was identified in 16 % (75/480) of isolates and pilus islet 2 (PI-2) in 3 % (12/480) of isolates, and 11 % (54/480) isolates were positive for both PI-1 and PI-2. PI-1 was found in 19 GPSCs and in both carriage and disease-causing populations. In contrast, PI-2 was only observed in disease-causing isolates belonging to five GPSCs (GPSC1, 2, 31, 32 and 96). Apart from one isolate in GPSC1, lineages GPSC1 (CC320) and GPSC84 (CC2975) carried both PI-1 and PI-2 pili.

**Table 3.** Six potential capsular switching events identified in the Indian dataset (*n*=480), 2009–2017

ST	Corresponding GPSC*	Serotype (*n*)	Description	Interpretation
90	GPSC23	6B (3) 6A (1)	Serotype 6B (ST90, CC385) isolates (*n*=3) from India are clustered together. The serotype 6A isolate from India is located in a clade predominantly expressing serotype 6B. It is most closely related to serotype 6B isolates from China and South Africa.	A capsular switching from serotype 6B to 6A occurred but it is not certain that it happened in India.
230	GPSC10	11A (1) 14 (1) 24 (1)	Serotype 11A (ST230, CC230) isolate from India is clustered closely with serotype 14 (ST230, CC230) from India, and they are grouped with 16 other serotype 14 isolates from South Africa in a serotype 14 sub-clade Serogroup 24 (ST8857, CC230) isolate from India is clustered with other serotype 24 isolates from elsewhere	A capsular switching possibly occurred from serotype 14 to 11A in India. Serogroup 24 variants possibly introduced into India from elsewhere rather than a recent capsular switching in India
320	GPSC1	19A (7) 19F (7) 9V (1)	Serotype 19A (ST320, CC320) and 19F (ST236, CC320) from India are clustered with other 19A and 19F isolates from elsewhere. Serotype 9V (ST320, CC320) is grouped in a 19F sub-clade and clustered with serotype 19F from India.	Serotype 19A and 19F variants possibly introduced into India independently from elsewhere rather than a recent capsular switching in India. A capsular switching possibly occurred from 19F to 9V in India
3735	GPSC30	10A (1) 10F (1)	The 10A (ST3735, CC4084) and 10F (ST3735, CC4084) isolates from Bangalore and India are clustered together with a 10F isolate from the USA in a clade that has a mixture of serotype 10A (*n*=10) and 10F (*n*=11).	Capsular switching between 10A and 10F is observed but its directionality is not certain.
5068	GPSC56	18B (1) 18C (10)	The serotype 18B (ST5068, CC5068) isolate is clustered with serotype 18C (ST5068, CC5068) isolates from India and Nepal (*n*=6)	A capsular switching possibly occurred from 18C to 18B in South Asia
9842	GPSC61	18A (1) 18C (1)	18A (ST9842, CC10689) and 18C (ST9842, CC10689) from India are clustered together.	A capsular switching possibly occurred between 18A and 18C in India

GPSC23 https://microreact.org/project/J1UXuuxFm/872be13e.

GPSC10 https://microreact.org/project/4V-kwgvLB/b56b0287.

GPSC1 https://microreact.org/project/bDQwV3W9FQMHfUgAAtHPuW/f2a35d94.

GPSC30 https://microreact.org/project/E_HCYxsYI/f9d198d6.

GPSC56 https://microreact.org/project/2zzqdMbjx/d76f9c38.

GPSC61 https://microreact.org/project/Yq6NyE3ZP/80ed26ca.

*GPSC-specific phylogenies are created using Indian isolates from this study and isolates from other countries in the GPS database (last accessed April 2019). These phylogenies can interactively be viewed along with geographical and temporal distribution at Microreact through the hyperlinks above.

### Antibiotic resistance

The predicted prevalence of antibiotic resistance in disease-causing and carriage populations is shown in Table 4. Disease-causing isolates had a higher prevalence of resistance to three beta-lactam antibiotics (penicillin, meropenem, cefuroxime) while chloramphenicol and cotrimoxazole resistance was higher among carriage isolates (Table 4). In both populations, VT had a significantly higher prevalence of resistance to all six beta-lactam antibiotics (penicillin, amoxicillin, meropenem, cefotaxime, ceftriaxone and cefuroxime) (Tables S2 and S3). In disease-causing populations, VT isolates also showed a higher prevalence in resistance to macrolide and cotrimoxazole and in multidrug resistance (Table S2). Among the non-PCV13 vaccine types in the disease-causing population, serotypes 24F, 10A and 15B showed multidrug resistance.

**Table 4.** Antimicrobial predicted resistance in disease-causing (*n*=294) and carriage (*n*=184) pneumococcal isolates from India, 2009–2017

	No. of isolates (%)		
Antibiotics*, †	Disease (*n*=294)	Carriage (*n*=186)	*P* value
Penicillin	149 (51)	53 (28)	<0.001‡
Amoxicillin	31 (11)	10 (5)	0.064
Meropenem	72 (24)	21 (11)	<0.001‡
Cefotaxime	60 (20)	29 (16)	0.228
Ceftriaxone	64 (22)	32 (17)	0.243
Cefuroxime	101 (34)	35 (19)	<0.001‡
Chloramphenicol	8 (3)	15 (8)	0.014‡
Erythromycin	143 (49)	80 (43)	0.260
Clindamycin	69 (23)	36 (19)	0.309
Cotrimoxazole	240 (82)	166 (89)	0.027‡
Tetracycline	160 (54)	110 (59)	0.345
Doxycycline	160 (54)	110 (59)	0.345
Multidrug resistance§	139 (47)	78 (42)	0.260

*Antibiotic resistance is predicted from genome data using a CDC pipeline tailored for *Streptococcus pneumoniae* (https://github.com/BenJamesMetcalf/Spn_Scripts_Reference) [15–17].

†No resistance to linezolid, levofloxacin, synercid, rifampin or vancomycin is detected.

‡Two-sided *P* values of <0.05 were considered statistically significant.

§Multidrug resistance (MDR) was defined as isolates resistant to ≥3 classes of antibiotics.

**Fig. 4.**
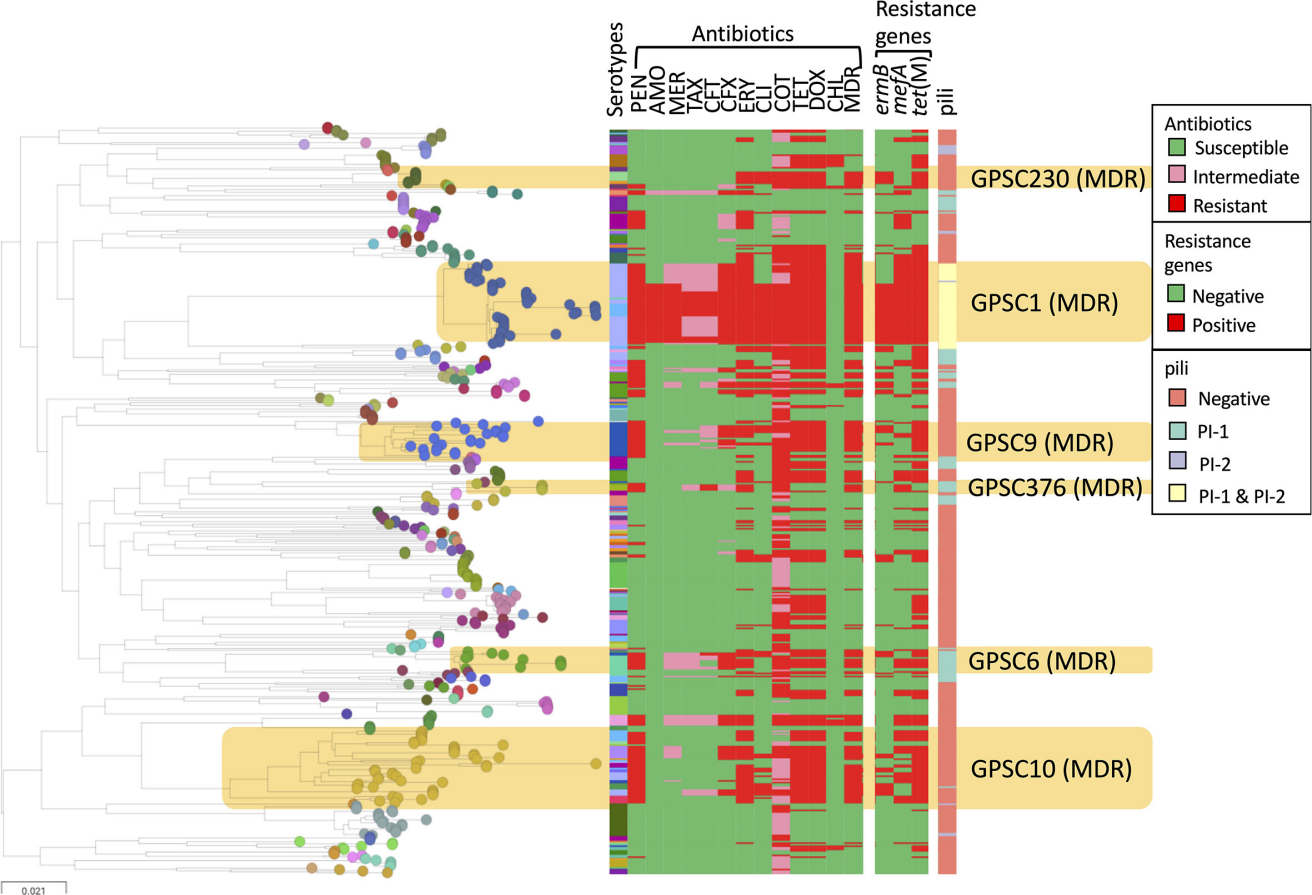
Maximum-likelihood tree reconstructed with all pneumococcal genomes in this study (*n*=480). The nodes of the tree are coloured according to the Global Pneumococcal Sequencing Clusters (GPSCs). Multidrug-resistant (MDR) lineages are highlighted and their corresponding *in silico* serotype, antibiotic-resistance profile and presence of pilus are indicated in the metablock. This figure can be visualized at https://microreactorg/project/GPS_India/2b522af9. PEN, penicillin; AMO, amoxicillin; MER, meropenem; TAX, cefotaxime; CFT, cefotaxime; CFX, cefuroxime; ERY, erythromycin; CLI, clindamycin; TET, tetracycline; DOX, doxycycline; CHL, chloramphenicol; MDR, multidrug-resistant.

Among pneumococcal lineages with more than five isolates, nine GPSCs (GPSC1, 6, 9, 10, 13, 16, 43, 91, 376) had >80 % isolates that were penicillin-resistant (minimum inhibitory concentration ≥0.12 µg ml^−1^). The majority of these lineages (GPSC6, 9, 10, 16 and 376) comprised both VT and NVT, two (GPSC1 and 13) were VT lineages and two (GPSC43 and 91) were NVT lineages (Fig. 3). Six multidrug-resistant lineages (*n*>5) are highlighted in Fig. 4; except for GPSC230, the other five lineages (GPSC1, 6, 9, 10 and 376) were penicillin-resistant (Fig. 4).

## Discussion

This study analysed the genetic lineages underlying both disease-causing and carriage isolates of pneumococci isolates in India pre-PCV introduction using a WGS approach. The predicted serotype distribution of the isolates based on the sequences of their capsular genes revealed a wide variety of capsular types. Of particular importance is the distribution of serotypes among children <2 years old, as 10-valent Pneumosil (Serum Institute of India) is being considered for expansion in the NIP to cover all children. Our data suggest that the existing (PCV13) and proposed vaccines (Pneumosil) for use in India will cover between 63 and 60 % of serotypes among children <2 years of age, respectively.

India is in the process of expanding the coverage of PCVs in its Universal Immunization Programme (UIP). In this study, 33 % (41/124) of invasive isolates recovered from blood and cerebrospinal fluid (CSF) were NVTs in children below the age of 5 years, which is slightly higher than a previous report of ~25 % NVT causing invasive disease in the same age group from Vellore, India [26, 27]. Similar to these previous reports, this study also observed an equal distribution of prevalence of NVTs, which makes it difficult to suggest any NVT for inclusion in future vaccine formulations based solely on prevalence. Among the NVTs observed in this study, some have high invasive disease potential, for example serotypes 2, 8, 12F and 24F. Serotypes 2, 8 and 12F are covered by PCV24 that is under development and there is also plan to include 24F in the future vaccine [28, 29].

Among the non-vaccine serotypes found in children, two rarely found serotypes were serotypes 2 and 45. Two serotype 2 isolates were identified to be causing bacteraemia, one from a child from Bangalore and one from an adult from Delhi, during 2015. Serotype 2 strains were common in adults a century ago and were rarely being identified as causing invasive disease [30, 19]. However, they have recently been described in meningitis cases among children from Bangladesh [31] and causing a widespread outbreak in Israel [32]. Similar to most of the serotype 2 isolates identified elsewhere, these two isolates belong to GPSC96 (CC74) [10]. The other rare serotype 45 found in India had the genetic background of ST3022 (GPSC245); this strain caused meningitis in an infant aged 5 months old. The same clone expressing serotype 45 was recovered from a CSF sample in Niger in 2006 [33][34], and a clonally related strain ST2212 (TLV of ST3022) causing meningitis was identified in Bangladesh during 2007–2013 [17, 35]. Serotype 45 was also found in other genetic backgrounds, for example ST3332 in The Gambia [36]. Serotype 2 and 45 were the 9th and 14th most common serotypes found in Gavi countries causing invasive pneumococcal disease among children under 5 years [37]. Therefore, they are potentially important serotypes to be considered for inclusion in future pneumococcal conjugate vaccines.

GPSC10 (CC230) is the only sequence cluster among the top five lineages in the disease-causing population to have both VTs and NVTs. It is the largest contributor of NVTs and accounted for 50 % of the major non-PCV13 serotype 15B/C in the disease-causing population [27], highlighting the potential of GPSC10 to mediate serotype replacement in the post-vaccine era. The NVT GPSC10 isolates expressing serotype 24F have an invasive disease potential similar to serotype 19A [24]. Increases in invasive diseases caused by serotype 24F pneumococci were also observed in Argentina (unpublished data), France [28] and Spain in children after the introduction of PCV13 [38]. In Spain, the increase was largely due to CC230 (major CC in GPSC10). GPSC10 is a multidrug-resistant lineage that is associated with resistance to penicillin, erythromycin, cotrimoxazole and tetracycline.

Antibiotic resistance is significantly higher among VT pneumococci, especially in disease-causing isolates. This finding suggests that the reduction of antibiotic resistance after the use of pneumococcal vaccines in developed countries could also occur in India via directly removing VTs that are associated with antibiotic resistance and via a reduction in febrile illnesses that often require antibiotic use [39, 40].

A limitation of this study is the relatively small sample size for each region, which does not allow us to investigate the potential differences in serotypes, strains and antibiotic resistance between regions during the phase introduction of the conjugate vaccines. To detect a 20 % difference in prevalence with 95 % confidence level, at least 196 samples are required for each region. However, to achieve this sample size may not be feasible due to the challenge of isolating pneumococci from suspected cases of pneumococcal diseases: antibiotic use prior to sampling and varied healthcare infrastructure in different regions. While obtaining a statistically sufficient number of disease isolates is not likely, cross-sectional studies sampling isolates from the nasopharynx from healthy carriers could be an alternative method to detect the impact of the vaccine.

This study, describing the pneumococcal disease and carriage epidemiology, demonstrates that 60–75 % of pneumococcal serotypes in children younger than 5 years is covered by PCV13 and Pneumosil. Vaccination against pneumococci is very likely to reduce antibiotic resistance. A multidrug-resistant pneumococcal lineage GPSC10 is a high-risk clone that could mediate serotype replacement. This study decsribes pneumococcal strain characteristics prior to vaccination that will help to evaluate changes associated with the NIP in the future.

## Supplementary Data

Supplementary material 1

Supplementary material 2
