# Supplementary material for: Streptococcus pneumoniae genomic datasets from an Indian population describing pre-vaccine evolutionary epidemiology using a whole genome sequencing approach
Source: Microb Genom. 2021 Sep 8;7(9):000645. doi: 10.1099/mgen.0.000645 (PMC8715438; doi:10.1099/mgen.0.000645)
Supplement: Supplementary material 1 [file mgen-7-0645-s001.pdf]

Legend: Figures

Figure S1: Distribution of non-PCV13 serotypes among invasive pneumococci from children <5yrs age group.

Fig S2: Serotype distribution in adult population (Age ≥50 years).

Legend: Tables

Table S1: The pneumococcal collection in this study by collection of year and clinical manifestation

Table S2: Antimicrobial predicted resistance of pneumococci expressing PCV13 and non-PCV13 serotypes in disease-causing pneumococcal isolates (n=294) from India, 2009-2017

Table S3: Antimicrobial predicted resistance of pneumococci expressing PCV13 and non-PCV13 serotypes in carriage pneumococcal isolates (n=184) from India, 2009-2017

Table S4: The distribution of non-PCV13 serotypes by age groups

Table S5: Serotype distribution among carriage and disease isolates from adults (≥ 50yrs of age)

Table S6: Fisher's Exact test data representing potential difference in serotype prevalence between children (age <5) and elderly (age>=50) in disease isolates

Table S7: Fisher's Exact test data representing potential difference in serotype prevalence between children (age <5) and elderly (age>=50) in carriage isolates

Table S1 The pneumococcal collection in this study by collection of year and clinical manifestation

Year	Disease	Carriage
2009	15	-
2010	16	-
2011	1	-
2013	10	12
2014	68	80
2015	63	1
2016	62	88
2017	31	1
2018	28	4
Total	294	186

Table S2 Antimicrobial predicted resistance of pneumococci expressing PCV13 and non-PCV13 serotypes in disease-causing pneumococcal isolates (n=294) from India, 2009-2017

	Number of disease-causing isolates (%)		
Antibiotics[a]	PCV13 serotypes (n=190)	Non-PCV13 serotypes (n=104)	P value
Penicillin	116 (61)	33 (32)	<0.001*
Amoxicillin	28 (15)	3 (3)	0.001*
Meropenem	59 (31)	13 (13)	<0.001*
Cefotaxime	55 (29)	5 (5)	<0.001*
Ceftriaxone	59 (31)	5 (5)	<0.001*
Cefuroxime	82 (43)	19 (18)	<0.001*
Chloramphenicol	6 (3)	2 (2)	0.717
Erythromycin	102 (54)	41 (39)	0.021*
Clindamycin	53 (28)	16 (15)	0.021*
Cotrimoxazole	168 (88)	72 (69)	<0.001*
Tetracycline	110 (58)	50 (48)	0.113
Doxycycline	110 (58)	50 (48)	0.113
Multidrug resistance[b]	99 (52)	40 (38)	0.0281*

[a]Antibiotic resistance is predicted from genome data using a CDC pipeline tailored for *Streptococcus pneumoniae* (https://github.com/BenJamesMetcalf/Spn_Scripts_Reference) (15-17, 31).
[b]Multidrug resistance (MDR) was defined as isolates resistance to ≥3 classes of antibiotics.
[c]No resistance to Linezolid, Levofloxacin, Synercid, Rifampin, Vancomycin is detected.
* Two-sided p values of < 0.05 were considered statistically significant.

Table S3 Antimicrobial predicted resistance of pneumococci expressing PCV13 and non-PCV13 serotypes in carriage pneumococcal isolates (n=184) from India, 2009-2017

	Number of carriage isolates (%)		
Antibiotics[a]	PCV13 serotypes (n=94)	Non-PCV13 serotypes (n=92)	P value
Penicillin	33 (35)	20 (22)	0.052
Amoxicillin	10 (11)	0	0.002*
Meropenem	21 (22)	0	<0.001*
Cefotaxime	25 (27)	4 (4)	<0.001*
Ceftriaxone	28 (30)	4 (4)	<0.001*
Cefuroxime	28 (30)	7 (8)	<0.001*
Chloramphenicol	5 (5)	10 (11)	0.188
Erythromycin	40 (43)	40 (43)	1
Clindamycin	20 (21)	16 (17)	0.579
Cotrimoxazole	80 (85)	86 (93)	0.096
Tetracycline	60 (64)	50 (54)	0.233
Doxycycline	60 (64)	50 (54)	0.233
Multidrug resistance[b]	40 (43)	38 (41)	0.883

[a]Antibiotic resistance is predicted from genome data using a CDC pipeline tailored for *Streptococcus pneumoniae* (https://github.com/BenJamesMetcalf/Spn_Scripts_Reference) (15-17, 31).
[b]Multidrug resistance (MDR) was defined as isolates resistance to ≥3 classes of antibiotics.
[c]No resistance to Linezolid, Levofloxacin, Synercid, Rifampin, Vancomycin is detected.
* Two-sided p values of < 0.05 were considered statistically significant.

Table S4. The distribution of non-PCV13 serotypes by age groups

	Disease (%)			Carriage (%)		
Age group (years)	n	Non-PCV13 serotypes nos (%)	Predominant Serotypes	n	Non-PCV13 serotypes nos (%)	Predominant serotypes
≤2	90	33 (36)	16F, 15B/C, 24, 10A, 17F, 20	9	3 (33)	11A, 13, 16F
3-5	48	12 (25)	15B/C, 10A, 8	42	16 (38)	7C, 11A, 16F, 23A, 34
6-24	20	4 (20)	15B/C, 34, 35A	37	12 (32)	6D
25-44	26	6 (23)	15B/C, 35B, 24, 33F, 11A, 8	31	17 (55)	22F, 11A, 28A, 31
45-65	63	27 (43)	15B/C, 8, 34, 10A, 15A	43	31 (72)	28A, 17F, 22F, 19B, 11A, 24, 31, 34
>65	46	21 (46)	24, 38, 15A, 35A, 35B/C	24	13 (54)	35A, 13

* Serotypes with a number of isolates equal or less than 1 were not listed.

Table S5: Serotype distribution among carriage and disease isolates from adults (≥ 50yrs of age)

	Carriage	Disease
Number of isolates	64	94
PPV23 Serotypes	31 (48.4%)	67 (71.2%)
Non-PPV23 serotypes	33 (51.6%)	27 (28.8%)
Predominant serotypes	19F, 17F, 28A, 3, 14, 11A, 35A, 13, 31, 34, 22F	19F, 14, 9V, 1, 3, 8, 19A, 24, 15A, 6A, 7F, 4, 23F

Table S6: Fisher's Exact test data representing potential difference in serotype prevalence between children (age <5) and elderly (age>=50) in disease isolates

Serotype	Manifest	Child	Elderly	Child_percent	Elderly_percent	p-value	Adjust_p value
14	Disease	10	6	7	6	1.00	1.00
23F	Disease	0	3	0	3	0.07	0.90
6B	Disease	8	1	6	1	0.09	0.90
36	Disease	1	0	1	0	1.00	1.00
24	Disease	4	4	3	4	0.72	1.00
19F	Disease	21	9	15	10	0.24	1.00
7B	Disease	1	0	1	0	1.00	1.00
10A	Disease	4	2	3	2	1.00	1.00
15B	Disease	4	2	3	2	1.00	1.00
19A	Disease	11	5	8	5	0.60	1.00
nspA_cps	Disease	0	0	0	0	1.00	1.00
6A	Disease	8	4	6	4	0.77	1.00
13	Disease	1	1	1	1	1.00	1.00
9V	Disease	4	6	3	6	0.32	1.00
11A	Disease	2	2	1	2	1.00	1.00
35B	Disease	1	2	1	2	0.57	1.00
15C	Disease	3	0	2	0	0.27	1.00
38	Disease	0	2	0	2	0.16	0.90
35A	Disease	1	2	1	2	0.57	1.00
6D	Disease	0	0	0	0	1.00	1.00
23A	Disease	0	2	0	2	0.16	0.90
18C	Disease	4	1	3	1	0.65	1.00
22F	Disease	0	1	0	1	0.41	1.00
48	Disease	0	0	0	0	1.00	1.00
19B	Disease	0	0	0	0	1.00	1.00
20	Disease	2	0	1	0	0.52	1.00
7F	Disease	4	4	3	4	0.72	1.00
12F	Disease	0	2	0	2	0.16	0.90
28A	Disease	0	1	0	1	0.41	1.00
17F	Disease	2	2	1	2	1.00	1.00
25F	Disease	1	0	1	0	1.00	1.00
40	Disease	0	1	0	1	0.41	1.00
18A	Disease	1	0	1	0	1.00	1.00
16F	Disease	4	1	3	1	0.65	1.00
6C	Disease	0	0	0	0	1.00	1.00
33B	Disease	0	1	0	1	0.41	1.00
18B	Disease	0	0	0	0	1.00	1.00
2	Disease	1	1	1	1	1.00	1.00
5	Disease	9	1	7	1	0.05	0.90

1	Disease	12	6	9	6	0.62	1.00
3	Disease	2	5	1	5	0.12	0.90
34	Disease	1	1	1	1	1.00	1.00
4	Disease	0	3	0	3	0.07	0.90
33F	Disease	2	0	1	0	0.52	1.00
9L	Disease	1	0	1	0	1.00	1.00
15A	Disease	0	4	0	4	0.03	0.90
45	Disease	1	0	1	0	1.00	1.00
10F	Disease	1	0	1	0	1.00	1.00
8	Disease	2	5	1	5	0.12	0.90
31	Disease	1	1	1	1	1.00	1.00
7C	Disease	0	0	0	0	1.00	1.00
28F	Disease	1	0	1	0	1.00	1.00
9N	Disease	0	0	0	0	1.00	1.00
25A	Disease	1	0	1	0	1.00	1.00
27	Disease	1	0	1	0	1.00	1.00

Table S7: **Fisher's Exact test data representing potential difference in serotype prevalence between children (age <5) and elderly (age>=50) in carriage isolates**

Serotype	Manifest	Child	Elderly	Child_percent	Elderly_percent	p-value	adjust_p value
14	Carriage	1	3	2	5	0.63	1.00
23F	Carriage	7	0	14	0	0.00	0.15
6B	Carriage	4	0	8	0	0.04	0.50
36	Carriage	0	2	0	3	0.50	1.00
24	Carriage	0	2	0	3	0.50	1.00
19F	Carriage	5	9	10	14	0.57	1.00
7B	Carriage	1	0	2	0	0.44	1.00
10A	Carriage	0	1	0	2	1.00	1.00
15B	Carriage	0	0	0	0	1.00	1.00
19A	Carriage	4	0	8	0	0.04	0.50
nspA_cps	Carriage	1	0	2	0	0.44	1.00
6A	Carriage	5	1	10	2	0.09	0.59
13	Carriage	2	3	4	5	1.00	1.00
9V	Carriage	0	1	0	2	1.00	1.00
11A	Carriage	3	3	6	5	1.00	1.00
35B	Carriage	0	1	0	2	1.00	1.00
15C	Carriage	0	1	0	2	1.00	1.00
38	Carriage	0	0	0	0	1.00	1.00
35A	Carriage	1	3	2	5	0.63	1.00
6D	Carriage	0	0	0	0	1.00	1.00
23A	Carriage	2	0	4	0	0.19	1.00
18C	Carriage	5	0	10	0	0.02	0.42
22F	Carriage	0	3	0	5	0.25	1.00
48	Carriage	0	1	0	2	1.00	1.00
19B	Carriage	0	2	0	3	0.50	1.00
20	Carriage	0	0	0	0	1.00	1.00
7F	Carriage	0	0	0	0	1.00	1.00
12F	Carriage	0	0	0	0	1.00	1.00
28A	Carriage	0	5	0	8	0.06	0.59
17F	Carriage	0	5	0	8	0.06	0.59
25F	Carriage	0	0	0	0	1.00	1.00
40	Carriage	0	1	0	2	1.00	1.00
18A	Carriage	1	0	2	0	0.44	1.00
16F	Carriage	3	0	6	0	0.08	0.59
6C	Carriage	1	0	2	0	0.44	1.00
33B	Carriage	0	0	0	0	1.00	1.00
18B	Carriage	0	0	0	0	1.00	1.00
2	Carriage	0	0	0	0	1.00	1.00
5	Carriage	0	2	0	3	0.50	1.00

1	Carriage	0	0	0	0	1.00	1.00
3	Carriage	0	4	0	6	0.13	0.78
34	Carriage	2	3	4	5	1.00	1.00
4	Carriage	1	0	2	0	0.44	1.00
33F	Carriage	0	0	0	0	1.00	1.00
9L	Carriage	0	0	0	0	1.00	1.00
15A	Carriage	0	2	0	3	0.50	1.00
45	Carriage	0	0	0	0	1.00	1.00
10F	Carriage	0	1	0	2	1.00	1.00
8	Carriage	0	0	0	0	1.00	1.00
31	Carriage	0	3	0	5	0.25	1.00
7C	Carriage	2	1	4	2	0.58	1.00
28F	Carriage	0	1	0	2	1.00	1.00
9N	Carriage	0	0	0	0	1.00	1.00
25A	Carriage	0	0	0	0	1.00	1.00
27	Carriage	0	0	0	0	1.00	1.00

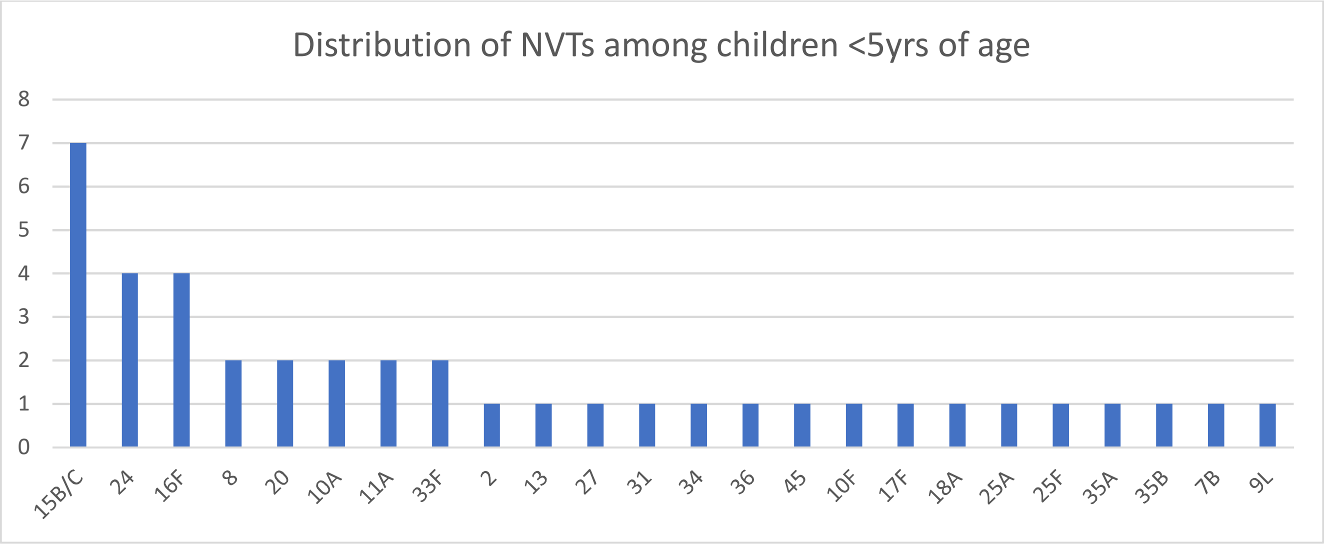

Figure S1: The distribution of non-PCV13 serotypes among invasive pneumococci from children <5yrs age group.

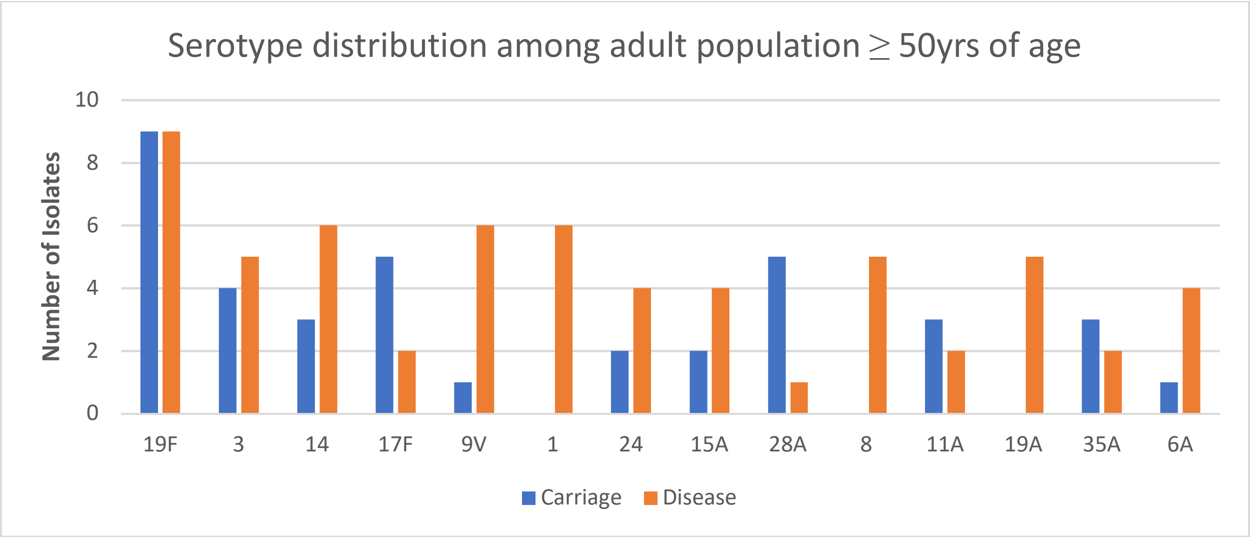

Fig S2: Serotype distribution in adult population (age ≥ 50yrs)
